# Construction of an instant structured illumination microscope

**DOI:** 10.1016/j.ymeth.2015.07.012

**Published:** 2015-10-15

**Authors:** Alistair Curd, Alexa Cleasby, Katarzyna Makowska, Andrew York, Hari Shroff, Michelle Peckham

**Affiliations:** a School of Molecular and Cellular Biology, Faculty of Biological Sciences, University of Leeds, Leeds, UK; b Section on High Resolution Optical Imaging, National Institute of Biomedical Imaging and Bioengineering, National Institutes of Health, Bethesda, MD, USA

**Keywords:** Construction, Fluorescence microscopy, Instant structured illumination microscope, Super-resolution

## Abstract

A challenge in biological imaging is to capture high-resolution images at fast frame rates in live cells. The “instant structured illumination microscope” (iSIM) is a system designed for this purpose. Similarly to standard structured illumination microscopy (SIM), an iSIM provides a twofold improvement over widefield microscopy, in *x*, *y* and *z*, but also allows much faster image acquisition, with real-time display of super-resolution images.

The assembly of an iSIM is reasonably complex, involving the combination and alignment of many optical components, including three micro-optics arrays (two lenslet arrays and an array of pinholes, all with a pitch of 222 μm) and a double-sided scanning mirror. In addition, a number of electronic components must be correctly controlled. Construction of the system is therefore not trivial, but is highly desirable, particularly for live-cell imaging.

We report, and provide instructions for, the construction of an iSIM, including minor modifications to a previous design in both hardware and software. The final instrument allows us to rapidly acquire fluorescence images at rates faster than 100 fps, with approximately twofold improvement in resolution in both *x*–*y* and *z*; sub-diffractive biological features have an apparent size (full width at half maximum) of 145 nm (lateral) and 320 nm (axial), using a 1.49 NA objective and 488 nm excitation.

## Highlights

• Instant structured illumination microscope: 2-fold resolution enhancement.
• Fast image acquisition rate (exceeding 100 fps).
• Detailed description of construction and use.

## Graphical abstract

**Figure:**
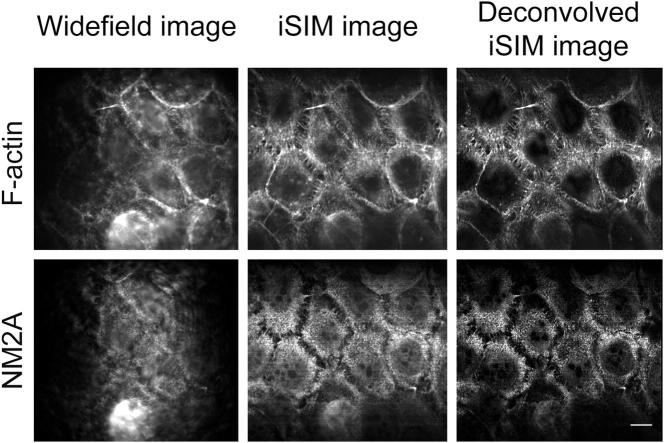

## 1 Introduction

Among the methods of acquiring super-resolution fluorescence images [1], [2], structured illumination microscopy (SIM) offers a relatively modest, twofold resolution improvement over widefield microscopy [3]. However, as SIM uses only a relatively small number of widefield images to capture the information required to improve resolution, it is in principle more suitable for live sample imaging; SIM offers the advantages of fast acquisition over a large area and weaker irradiation of the sample compared to alternative techniques such as stimulated emission depletion [4] and single-molecule localisation [5], [6], [7], and it is compatible with all fluorophores used in widefield and confocal imaging.

In SIM, the combination of spatial frequency components from an illumination beam and the sample itself allows a doubling of the maximum spatial frequency theoretically obtainable in the image, compared with widefield techniques [3]. However, to extract the high spatial frequency information, the sample must be imaged multiple times, using different orientations of the patterned illumination, and the final image is only obtained once these images have been processed. Moreover, in practice, some of the theoretical frequency space is not accessed. Nonlinear variants of SIM [8], [9] can introduce still higher spatial frequencies to the combination of illumination and fluorescent response by saturation of the fluorescence or of the dark state of a switchable fluorophore (e.g. Dronpa). SIM can also be used to provide depth-sectioning with an excitation pattern structured in three dimensions [10].

The basis of a confocal microscope is the combination of diffraction-limited illumination and detection through a pinhole, which results in a narrower point spread function (PSF) than using either pinhole detection or diffraction-limited illumination alone. The confocal PSF is the product of the emission and excitation PSFs. If the emission pinhole is made very small, then the confocal PSF will be narrower, but there is a detrimental reduction in signal, obscuring the resolution gained in principle. By scanning a displaced pinhole, and appropriately shifting and summing the resulting images, much more of the emission signal can be used, and the narrower PSF retained; the appropriate shift is ½ the pinhole displacement for each image. This principle was first discussed in 1988 [11], and further developed into “image scanning microscopy” [12], in which a focussed excitation laser was scanned over the sample, and an image taken at each scanning focus position. The raw images were then scaled by a factor of 0.5 and combined to generate the full image. Deconvolution of the resulting image resulted in a narrowing of the PSF of the modified confocal microscope to 150 nm (2σ width of a Gaussian fit). However, the process of scanning and capturing an image at every position was very slow, and not suitable for live cell imaging.

The speed of this type of imaging was improved to several frames per second in the MSIM (multifocal SIM) by using a digital mirror device (DMD) to generate multiple excitation foci, which were scanned across the sample [13]. The foci were organised into a sparse pattern, and a fluorescence image was acquired each time the pattern was translated by the equivalent of one DMD pixel (120 nm when imaged at the sample), until the entire field had been scanned. Each acquired image was processed in software such that the fluorescence from each excitation spot was pinholed (set to zero beyond a chosen radius, to reject out of focus light) and scaled by 0.5 before summing all images to provide one frame.

**Fig. 1:**
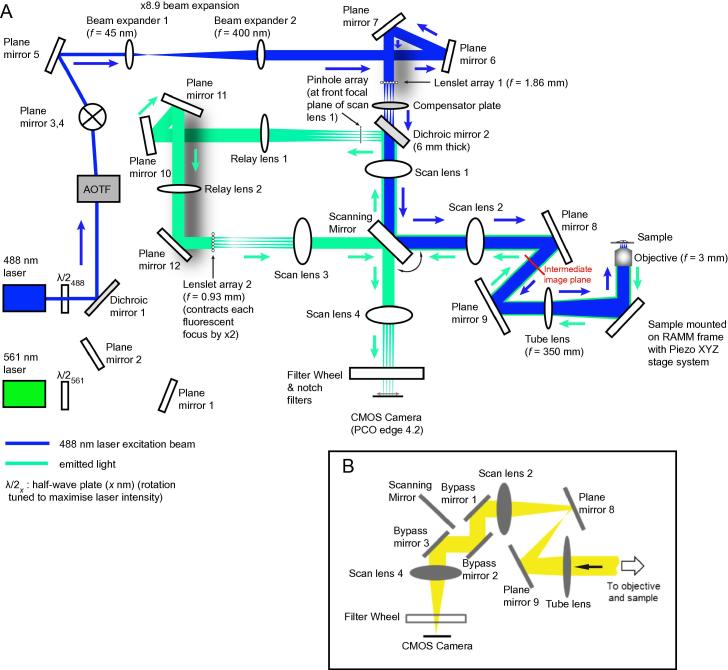
The layout of the implementation of iSIM at UoL, designed to replicate the function of the original [14]. (A) The overall set-up for the iSIM. Plane mirrors 3 and 4 form a periscope to raise the beam height to match the axis of the sample stage. The beamlets generated by lenslet array 1 are focussed at the sample, and scanned across it by the scanning mirror, which rotates about the *y*-axis as indicated. The beamlets also come to a focus at the intermediate image plane shown. (B) The positions of bypass mirrors implemented when imaging in brightfield. The majority of the hardware was fixed to a metric anti-vibration table (Table 1). 1″ diameter pedestal posts and fork clamps were used to attach most components to the table, with component height adjustable using spacers or ½″ diameter post holders and posts (Table 1).

The “instant structured illumination microscope” (iSIM) [14] is a further development, but uses optical hardware (an “analogue” approach), to perform the shifting and summing, enabling the resulting higher-resolution images to be visualised “instantly”. A set of beamlets are generated by a lenslet array (lenslet array 1, Fig. 1) and focussed onto a sample. The resulting fluorescence image is passed through a set of pinholes to reject out of focus light, and then passed through a second lenslet array (lenslet array 2, Fig. 1) that focusses the beamlets, such that each image from an excitation spot is scaled by a factor of 0.5. As a scanning mirror (Fig. 1) scans the excitation across the sample, and also scans the resulting images across the camera, the resulting fluorescence images are integrated in a single exposure, and immediately displayed. More detailed explanation of how the optical design achieves the narrower PSF by image scanning and shifting can be found in Supplementary note 1. The resolution is increased (a reduction in the full width at half maximum, FWHM, of the PSF) in these real-time images by a factor of √2 compared with a widefield microscope, and the final improvement is a factor of 2, following image deconvolution using the measured PSF of the optical system. Image capture is very rapid (potentially exceeding 100 frames per second), making such a system useful for observation of dynamic processes in live cells.

We chose to build an iSIM at the University of Leeds (UoL), in order to take advantage of its speed and resolution, and its compatibility with standard sample and fluorophore properties, and this chapter is aimed at providing detailed methods for those who wish to build a similar instrument.

## 2 Materials and methods

### 2.1 Design of the iSIM

In essence, the iSIM we constructed at Leeds was based on the published iSIM already described, and we refer readers to that publication for a detailed explanation of the iSIM and its underlying theory [14]. Briefly, in the iSIM, excitation illumination is provided by two 1 W lasers, which have a wavelengths of 488 and 561 nm (Table 1, Fig. 1). Their paths are combined at a dichroic mirror (Fig. 1), and deflected into the system as required by the acousto-optic tunable filter (AOTF); only one excitation beam at a time enters the rest of the system. Half-wave plates *λ*/2_488_ and *λ*/2_561_ rotate their polarisation for optimal transmission into the system path by the AOTF.

A periscope provided by plane mirrors 3 and 4 raises the beam to the correct height for entry into the sample stage. The beam is then expanded by passing through two lenses such that it covers a larger area of the first lenslet array (*f = *1.86 mm), a converging microlens array which generates a multifocal excitation pattern. The light emerging from this array is passed through a tilted compensator plate, which gives rise to an astigmatism orthogonal to, and thus compensating for, that introduced by the 6 mm thick dichroic mirror (dichroic mirror 2) in the diverging beamlets.

The light then passes through a pair of scan lenses (scan lens 1 and 2), which act as a 1:1 telescope (Fig. 1). Between these lenses, placed at their focal points, is the scanning mirror, which scans the excitation beam across scan lens 2. The scanned excitation pattern is demagnified and focussed on the sample by the tube lens (*f = *350 mm) and the objective (*f *= 3 mm). The long focal length of the tube lens also contributes to ensuring sufficient sampling at the camera and decreases vignetting on the emission path at the scanning mirror (by demagnifying the objective back focal plane in combination with scan lens 2).

Emitted fluorescence light from the sample is collected by the objective and transmitted back to dichroic filter 2, via the tube lens, scan lens 2, scanning mirror and scan lens 1 (Fig. 1). The scanning mirror thus descans the fluorescence beamlets returning from the sample. At dichroic mirror 2 the beamlets are reflected towards the pinhole array (see Section 2.2.1), which rejects out of focus fluorescence. The pinhole array is aligned with the first lenslet array, such that it is conjugate with the focussed excitation pattern. Light emerging from the pinholes is passed through relay lenses 1 and 2, which relay the fluorescence image pattern to the second lenslet array. This is aligned with both with the first lenslet array and the pinhole array (see Section 2.2.1). In this part of the light path, there are several plane mirrors (10, 11 and 12); these ensure an appropriate (even) total number of lateral image inversions between the first and second sides of the scanning mirror, such that re-scanning of the final image across the camera is carried out in the correct direction. On passing through the second microlens array, each pinholed fluorescent emission image is contracted by a factor of 2, before passing through the first of a second set of scan lenses (scan lenses 3 and 4). Between these scan lenses, the scanning mirror is again positioned at their focal points. The scanning mirror scans the scaled fluorescence images across the sCMOS (scientific complementary Metal-Oxide-Semiconductor) sensor of the camera. One or more scans of the sample and the fluorescence pattern are collected in a single camera exposure, which results in the final image (before any image deconvolution in software).

A filter wheel (Fig. 1) is used as necessary to block light at undesired wavelengths. Two notch filters are used (in the same filter wheel port) to filter stray excitation light, while allowing maximum transmission of the fluorescence beams. For samples with some combinations of fluorophores and labelling densities, bandpass filters may be required to separate the colour channels [14]. Other filter combinations should contain the same overall optical path length as this double notch filter combination (4.0 mm through fused silica).

A Python script (*camera_display.py* and its dependencies, available at https://code.google.com/p/msim/downloads/list^1^) controls the electronic elements of the iSIM and provides a graphical user interface, after minor modifications.

### 2.2 Set up procedure

#### 2.2.1 Optical arrangement

The following steps were found to be an efficient method for achieving alignment, minimising the repetition of work on the same section of the optical system.

##### 2.2.1.1 Choose the overall position of the system

Place the scanning mirror in its mount near the centre of the table; set rough positions for the rest of the system, based on combinations of focal lengths of the components (Table 1 and described below). Plane mirrors 8 and 9 give some flexibility to the positioning of the RAMM stage. Positioning of all the components in the system before lenslet array 1 is flexible (apart from the length between the two beam expander lenses). The distance between relay lenses 1 and 2 is also flexible.

##### 2.2.1.2 Align the path from scan lens 1 to the sample

The scanning mirror needs to be at the focal point of scan lens 1, i.e. the front focal length from the end of its housing (the front focal length is different from the focal length of 190 mm, which is defined from an unmarked plane within the lens system). The front focal length of the scan lenses was measured to be 170 mm, using autocollimation with a 10 μm pinhole.

To set this distance, use the auxiliary collimated diode laser (Table 2) to input a collimated beam to scan lens 1 (from above it in Fig. 1) such that it is undeviated by the lens. (You should check that introducing the lens does not change the beam position beyond it.) Focus this beam onto the centre of the useful scanning mirror area. Make this auxiliary beam collimated (test with the shearing interferometer, Table 2) and undeviated when scan lens 2 is introduced. (The excitation beam could be used for this – reversing steps 2 and 3 of this procedure – but it is simpler to align the components of this step without the need to choose the beam alignment with plane mirrors 6 and 7.)

Remove the tube lens and objective lens and adjust plane mirrors 8 and 9 so that the same auxiliary beam (collimated by scan lens 2) now passes through the stage, centred on its input and output (objective mounting) apertures, and so that it exits vertically from the output aperture. It may be helpful to mark a position where the beam is incident on a surface above the stage. Replace the tube lens and objective, and align the tube lens so that the smallest beam spot possible is formed on a surface directly above the objective. The tube lens is now set to provide the correct tube length, and aligned with the objective.

Now, to set the optical path length between scan lens 2 and the tube lens (540 mm), position the auxiliary laser such that a collimated beam is incident on scan lens 2, from the scanning mirror side. An auxiliary mirror will also be necessary to fold the auxiliary beam into the correct path, between scan lenses 1 and 2; two positions along the beam should be marked before moving the auxiliary laser so that the beam can be aligned to the same path, with the new laser position. Reposition the plane mirrors 8 and 9, such that the beam is collimated after the tube lens (an auxiliary mirror is again necessary, between the tube lens and the RAMM stage, to fold the beam, allowing access to it with the shearing interferometer). Check whether the beam is incident on the centre of the input aperture to the stage; if necessary, adjust the beam position on this aperture using plane mirrors 8 and 9 and repeat, again testing for collimation after the tube lens. Now, with a collimated beam incident on scan lens 1, the small beam spot should be visible in the same location directly above the objective lens.

##### 2.2.1.3 Align from the excitation lasers to scan lens 1 (lenslet array 1 not in place)

Use plane mirrors 1 and 2 to make the 561 nm beam collinear with the 488 nm beam. Pick two positions along the beam path where they must be coincident, and use an auxiliary mirror to provide a longer beam path without expanding the beams. Maximise the modulation efficiency of the AOTF by using its remote control to set the frequency and amplitude of crystal vibrations to maximise diffraction efficiencies for the 488 nm and 561 nm beams in the channels chosen for them at the AOTF connector (channel choice at the AOTF does not matter, except that the AOTF channels must be connected to the correct outputs from the computer, see Section 2.2.2). Next, use the half-wave plates (*λ*/2*_x_*, Fig. 1) to maximise diffraction efficiencies for each of the two wavelengths. Use an optical power meter (Table 2) to measure the diffracted power.

Next, using the 488 nm excitation beam, align beam expander lens 1 so that the beam passes through it undeviated. Align beam expander lens 2 (beam expander separation: 445 mm) so that the beam leaving it is collimated and undeviated. Align plane mirrors 6 and 7 such that the small beam spot is observed directly above the objective lens, without lens array 1 in place. Check collimation after plane mirror 7 and adjust beam expander 2 if necessary. We found that collimation was affected by components from plane mirror 6 to the compensator plate, presumably by some curvature of an optical surface.

##### 2.2.1.4 Align from scan lens 1 to the camera (emission path), without pinhole array and lenslet array 2 in place

Make the auxiliary collimated laser beam incident on scan lens 1 from the scanning mirror side. Align relay lens 1 so that the beam is undeviated and collimated once it has passed through it (optical path from scan lens 1 to relay lens 1: 490 mm).

Position scan lenses 4 and 3 using the same method as for scan lenses 1 and 2 (without the camera and filter wheel FW in place), with the auxiliary beam incident first on scan lens 4. Mark the beam position at two locations along its path after scan lens 3.

Move the auxiliary laser so that the beam is now incident on scan lens 3 from the scanning mirror side, following the same marked path. Align relay lens 2 so that the beam is undeviated and collimated once it has passed through it (optical path from relay lens 2 to scan lens 3: 490 mm). Mark the beam path after it passes through relay lens 2.

Move the auxiliary laser so that it is incident on relay lens 2 from the side nearest scan lens 3, following the marked path. Adjust plane mirrors 11 and 10 so that the auxiliary beam is collinear with the 488 nm excitation beam at dichroic mirror 2 and another location (e.g. above the objective lens, or on a lens or mirror). Since the distance between the relay lenses is flexible, the auxiliary beam does not need to be focussed or collimated at the same positions as the excitation beam when making the two beams collinear.

##### 2.2.1.5 Array elements step 1 (align lenslet array 1 and pinhole array)

Position lenslet array 1 approximately 123 mm from the rear of the housing of scan lens 1 (this distance is the back focal length of the scan lenses minus the extra optical path through the silica of the thick dichroic mirror 2 and compensator plate).

Send the collimated auxiliary beam through scan lens 1 from the scanning mirror side (coincident with the 488 nm beam at two points). Insert and position the pinhole array so that the beam is collimated after relay lens 1 (optical path from pinhole array to relay lens 1: 300 mm).

**Fig. 2:**
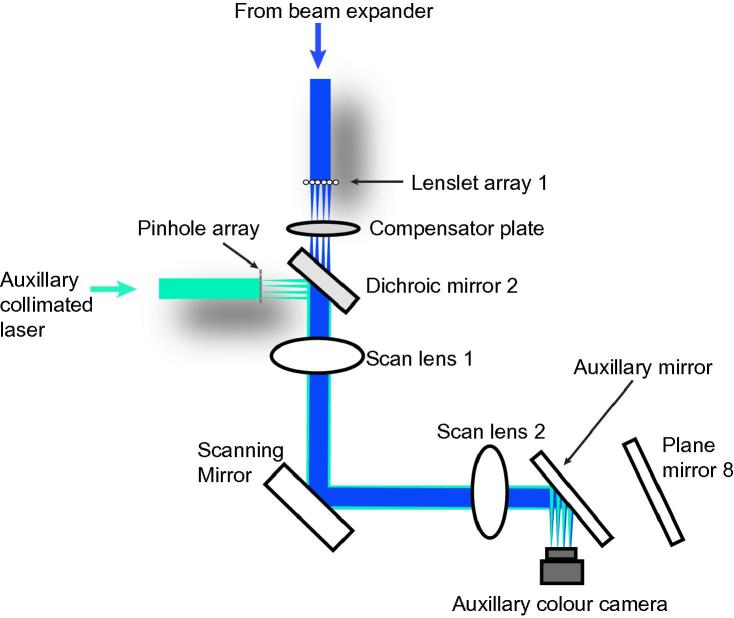
Use of an auxiliary laser beam, mirror and camera, together with the excitation beam, to align the foci of lenslet array 1 to the pinhole array. (See Fig. 1 for context.)

To align lenslet array 1 to the pinhole array, illuminate the pinhole array from the side nearest relay lens 1 with the auxiliary laser, and illuminate lenslet array 1 with the 488 nm excitation beam. Place the auxiliary camera in an intermediate image plane (see Fig. 2) such that images of the pinholes (green) are in focus. Rotate and translate lenslet array 1 such that images of the foci of this array (blue) are also in focus and concentric with the images of the pinholes.

##### 2.2.1.6 Position camera

The focal plane specified within the camera should be 128 mm behind scan lens 4 (equal to the back focal length of the scan lens minus the extra path length through the notch filters). The back of the blue counter ring on the F-mount adapter on the PCO edge 4.2 should therefore be 111 mm behind scan lens 4, from drawings of the camera design, supplied by PCO. Illuminate the objective from the sample side using the brightfield source. With the filter wheel and notch filters in place, but without lenslet array 2 in place, use the translation stage to finely adjust the position of the camera to obtain clear, round images of the pinholes at the camera. These images can be viewed on a monitor using the PCO CamWare software supplied with the camera.

##### 2.2.1.7 Array elements step 2 (align lenslet array 2 and pinhole array)

Position lenslet array 2 approximately 129 mm behind scan lens scan lens 3 (the scan lens’ back focal length). With the pinhole array in place, illuminate the objective from the sample side, and rotate and translate (in *x*–*y*) lens array 2 until the array of image spots (viewed in CamWare) covers the largest area possible on the camera sensor. When the pinhole array and lenslet array 2 are not aligned, the array of spots formed on the camera sensor covers a smaller field of view. Using a fluorescent sample, illuminated with one of the excitation lasers, move lenslet array 2 in *z*, to form images half the size of the pinholes, or as close to this as possible, on the camera sensor (viewed in CamWare). We used clusters of fluorescent beads (4 μm diameter) for this step (Life Technologies, TetraSpeck Fluorescent Microspheres Size Kit T14792, Wells 1 or 6), but other bright fluorescent samples (see next step) can be used.

##### 2.2.1.8 Array elements step 3 (rotation angle of the arrays)

Use a fluorescent sample (we used a thin, uniform fluorescent layer [14], e.g. a dilute solution of Alexa Fluor 488 labelled antibodies, or cells stained with Alexa Fluor 546 phalloidin, both from Life Technologies). Without lenslet array 1 or the pinhole array in place, take iSIM images, rotating lenslet array 2 to minimise the stripes visible on the monitor. Scanning the angled array of emission spot images across the camera sensor causes these stripes. Capturing a 2D (single *z*-slice) time-lapse sequence with ∼2 s between frames is helpful during this alignment step, with the live iSIM images appearing on a monitor.

##### 2.2.1.9 Array elements step 4 (lenslet array 2 and pinhole array)

Repeat step 7, but leave the position of lenslet array 2 as set in step 8 and rotate and translate (in *x*–*y*) the pinhole array, to align it with lenslet array 2 and obtain images of the large area array of demagnified emission spots.

##### 2.2.1.10 Array elements step 5 (lenslet array 1 and pinhole array)

Using the auxiliary camera as in step 5, realign lenset array 1 to the pinhole array.

Care must be taken when removing and replacing the array elements; a slight mechanical jarring (e.g. when contact between the mount and another surface can be clearly heard) can disturb the alignment of the arrays in their mounts. Realignment of the array elements may therefore be necessary if they are removed and replaced.

To maximise contrast of the images, it is necessary to filter stray light from the system. Screens and apertures can be introduced as required to prevent stray light from arriving at the camera chip. In particular, an aperture close to the scanning mirror is required, between scan lens 1 and the scanning mirror, to prevent light from travelling past the edges of the scanning mirror and being focused onto the camera chip by scan lens 4. Stray light can be identified as background pixel values in CamWare, and in the final iSIM images.

#### 2.2.2 Electronic control

The AOTF, scanning mirror, piezoelectric sample stage and camera were controlled from the computer, using the analogue output card and breakout box (Table 1). This iSIM implementation uses a different analogue output card from that in the previously published system [14], and does not require the home-built current amplifiers used in that case.

Three channels require connecting from the analogue output card (via the breakout box) to the AOTF: voltages for controlling the 488 nm laser beam, the 561 nm laser beam and the “blanking” channel, which switches off (or allows switching on) all channels of the AOTF as required (e.g. between exposures). Using *camera_display.py* for control, these are channels 3 (blanking), 4 (controlling the 488 nm laser) and 5 (controlling the 561 nm laser), at the analogue output card.

The position control connection for the scanning mirror connects to channel 0 of the analogue output card, using *camera_display.py*.

Control of the piezoelectric sample stage was through channel 1 of the analogue output card, using *camera_display.py*.

The sCMOS camera was connected to the workstation using its accompanying data acquisition card; a trigger signal to acquire an exposure was supplied by the analogue output card (channel 2, using *camera_display.py*).

*camera_display.py* controls the filter wheel and interrogates the sample stage controller using serial communication. The filter wheel was controlled by serial over USB communication, as per the standard installation procedure (using driver SI_CDM_v2.10.00 from the manufacturer). The baud rate used by the filter wheel controller in communication with the computer was changed at the controller to 9600, to match the serial over USB rate. The sample stage controller was interrogated for sample position through a USB to UART Bridge Virtual COM Port (installed and run using driver CP201x, Silicon Laboratories Inc., TX).

#### 2.2.3 Software adaptation and use

Some minor changes may be required to the downloadable scripts, before they control the system as designed, since some parts of the code are specific to the hardware being used. The scripts must also be on the search path used by Python. For the replica iSIM, changes to the following were made:

• *board_name*, on line 13 in *ni.py* (changed to “DAQ-NI-67633”, to match the output card).
• The maximum data rate allowed on line 40 in *ni.py* (changed to 740000, to match the output card). The user warning immediately following was also changed to match.
• The port number used by *serial.Serial* on line 7 in *sutter.py* (changed to 2, to match the installation of the filter wheel on port COM3).
• The port number used by *serial.Serial* on line 6 in *xyz_stage.py* (changed to 3, to match the installation of the sample stage on port COM4).
• *pco_edge_type* on line 16 in *pco.py* (changed to 4.2, to match the camera model).
• The data folder for image acquisition was changed on line 2101 in *camera_display.py* (from “D:////amsim_data” to “C:/iSIM_data”).
• References to icons for cosmetic use in the user interface were commented out, i.e. lines 226–228 and 894 in *camera_display.py* (icons must be supplied to execute these lines).

For use of the analogue output card, manufacturer-supplied DLLs are required to be either on the Python search path, or in the same directory as the hardware control scripts. For this hardware, these DLLs are *nicaiu.dll* for the analogue output card, and *sc2_cl_me4.dll* and *SC2_Cam.dll* for the sCMOS camera.

Running *camera_display.py* begins control of the iSIM.

A new installation of Python 2.7.8 64-bit, with additional packages *setuptools* 5.8, *numpy* 1.8.2, *scipy* 0.14.0, *matplotlib* 1.4.2, *ipython* 2.3.1, *pyserial* 2.7 and their dependencies, executed the code as expected. The additional packages were installed using installers freely available at http://www.lfd.uci.edu/~gohlke/pythonlibs/.

A downloadable Python distribution and environment (Enthought, Canopy) was tested as a potential user-friendly Python installation, but it did not handle some packages as expected by the code.

### 2.3 Image acquisition

#### 2.3.1 Brightfield imaging

Initially, the brightfield mode of the iSIM allows the user to find a region of interest for super-resolution fluorescence imaging. When in brightfield mode, bypass mirrors are used to transmit light from the sample to the camera without passing through the pinholes and lenslet array 2 (Fig. 1B). Bypass mirror 2 is constantly in position, and bypass mirrors 1 and 3 are on flipping mounts (Thorlabs, MFF101/M) which are connected to the analogue output card (channel 6, using camera_display.py). A BNC T-junction (Radio Shack, 616-3017) connects both flipping mounts to the same cable for connection to the analogue output card via the breakout box.

#### 2.3.2 iSIM acquisition

**Fig. 3:**
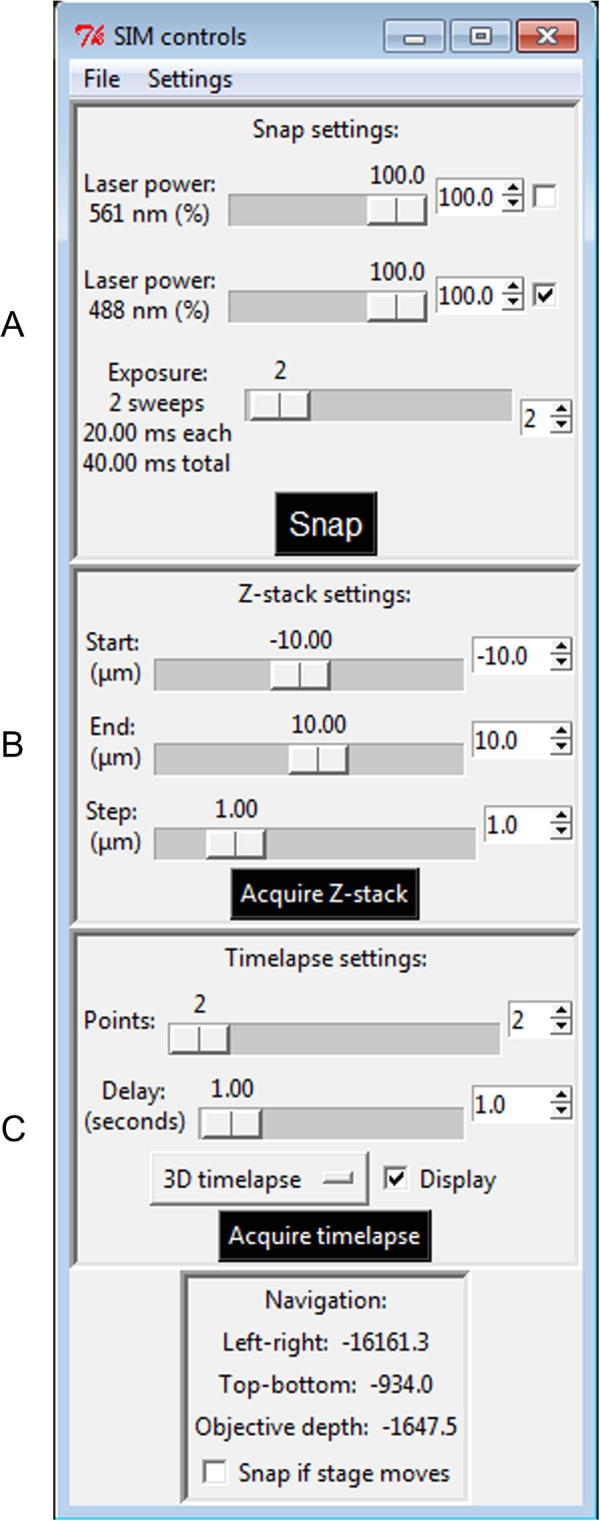
The graphical user interface for iSIM control. (A) Selection and control of the excitation laser and laser power, and number of scanning mirror sweeps per exposure; snapshot button. (B) *z*-Stack settings for the start and the end of the *z*-stack, and the step size required, together with the “Acquire Z-stack” acquisition button. (C) Timelapse settings controlling the number of images required, and the delay between the images, together with the “Acquire timelapse” acquisition button. Below this panel is a check box “Snap if stage moves” which can be checked when scanning the sample, for continuously previewing the sample.

Imaging is controlled by a simple graphical control interface, generated by *camera_display.py* (Fig. 3). Exposure of the specimen to light can be controlled by adjusting the transmission efficiency of each excitation beam into the system using the AOTF, and by varying the number of sweeps of the scanning mirror to be integrated into the exposure (Fig. 3A). As the system does not have eyepieces, regions of interest on the sample must be found using the live iSIM display, while moving the stage with the joystick and *z*-adjustment wheel, and acquiring “Snap” images (which are not saved to disk). By employing the command “Snap if stage moves”, snap images are captured automatically during this process, with images refreshed for each new stage position. This searching process should use reduced excitation power, and the most appropriate excitation wavelength to minimise photobleaching of the sample. The optimum start and end points for a *z*-stack can then be chosen using the “Acquire Z-stack” command (Fig. 3B), using large step sizes (0.5 μm or greater) to minimise bleaching. For the final image acquisition, step sizes in *z* should be 0.25 μm or smaller, to contain sufficient information for subsequent image deconvolution (see Section 2.3.3). Setting the “Start” value for the *z*-stack (Fig. 3B) to 0 μm or greater, and the “End” value to greater than the “Start” value, avoids any reversal in the direction of travel for the sample stage during capture of the *z*-stack, which provides the best image stability.

When images are acquired with “Acquire Z-stack” or “Acquire timelapse” (Fig. 3C), they are saved as 16-bit files in TIFF format. A *z*-stack is saved as a single file, containing the multiple slices, while for a time-lapse sequence, one file per time-point is saved, as required for image deconvolution (see Section 2.3.3). Acquisition parameters are saved in accompanying text files with the same name. If different emission filters are used for the two colour channels, each time-point or stack in the output is separated into two files. Following acquisition, *ImageJ* [15] opens the TIFF files automatically for all *z*-stacks. It will also do this for time-lapse sequences, but only if “Display” (Fig. 3C) is checked. At this point, time-lapse frames are combined into a single hyperstack so that the (pre-deconvolution) sequence can be viewed (this hyperstack is not automatically saved).

The raw images acquired, and displayed in real time, by an iSIM have resolution improved by a factor of √2, compared with widefield microscopy using the same optics [14] (see also Section 3). Every frame appears after capture on the live iSIM display during a time-lapse sequence.

The “Settings” menu allows the user to access the brightfield mode of the microscope (see Section 2.3.1), choose filter wheel positions for the two colour channels, and adjust other display and acquisition settings. It may be necessary to tune “Galvo mirror DAQ points per sweep” in “Settings”, “Galvo mirror”, to avoid diagonal striations in the image. For the UoL iSIM, this parameter is set to between 180 and 220.

#### 2.3.3 Image deconvolution

Since the support of the optical transfer function of the system is theoretically doubled by the iSIM contraction and scanning mechanism [11], [14] (Supplementary note 1), a full twofold improvement in resolution (halving the width of the PSF compared with the widefield case) is possible with a suitable deconvolution algorithm. Deconvolution of raw iSIM images with the PSF found in raw iSIM images does result in this recovery of information [14] (see also Section 3). The raw PSF can be obtained from measurements of images fluorescent beads [14], or from those of small (<100 nm) fluorescent features in a sample.

A deconvolution program, *decon.py*, is included in the downloadable iSIM software. This program can receive either a user-supplied three dimensional PSF (in TIFF or RAW formats) or numerical input of FWHM in the three dimensions (in pixels), assuming a Gaussian PSF.

An alternative is the use of the *ImageJ* plugin *DeconvolutionLab* (Biomedical Imaging Group, EPFL, Switzerland), with PSFs generated, for instance, using the plugin *Gaussian PSF 3D* (OptiNav). The same algorithm as used in *decon.py* (Richardson-Lucy) is available, and images can be batch processed. (Each image must be a one-channel *z*-stack; the program interlaced images from the two channels when asked to deconvolve a two-colour *z*-stack.)

For time-lapse sequences, both programs require one file input per frame, since they are designed for single images (2D or 3D). When provided with multiple time-points in one file, as can be viewed and saved in an *ImageJ* hyperstack, the software will appear to process the images, but successive frames will be concatenated in the *z*-direction, resulting in an incorrect image before processing.

York et al. [14] found that commercial software (Huygens, Scientific Volume Imaging) performed better than *decon.py* for 2D (single *z*-slice) iSIM images.

## 3 Results and discussion

### 3.1 Spatial image characteristics and resolution

The default field of view (FOV) of the UoL iSIM is 90 μm × 80 μm (1745 × 1550 pixels); lateral spatial sampling at the specimen is 56.0 nm per pixel (it was designed to be 55.5 nm [14]). The *y*-extent of the FOV can be increased further through “Settings”, “Camera ROI”, but with little benefit to the image (see Section 3.3). The FOV can also be decreased to decrease the minimum time between frames in time-lapse imaging (see Section 3.2). A plan objective is required to fill the default FOV with a high quality image. The minimum step size between *z*-slices that the user can select is 0.05 μm.

**Fig. 4:**
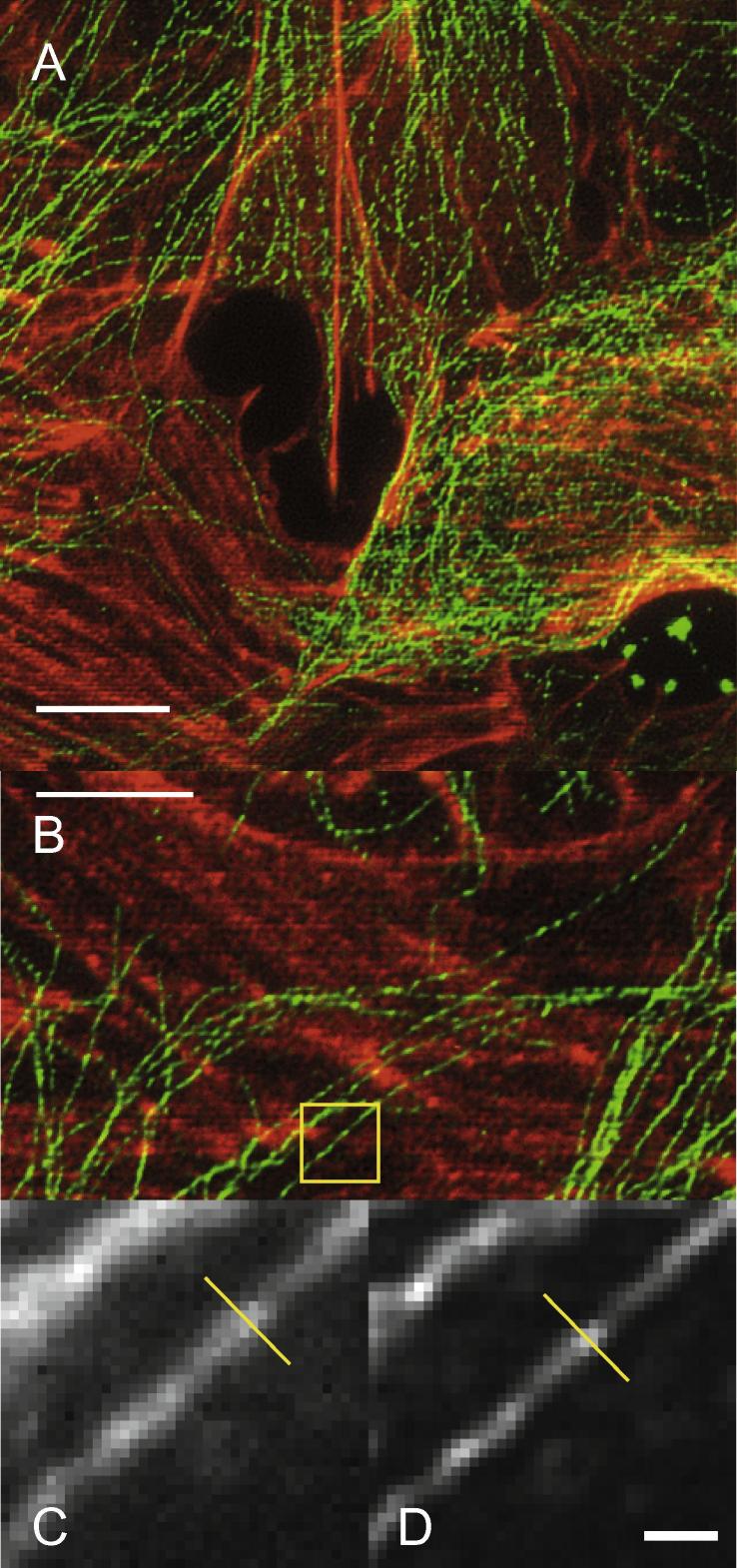
iSIM images of cells co-stained for F-actin and microtubules. (A, B) Maximum intensity projections in *x*–*y* from 80 *z*-slices taken over a 4 μm depth (scale bars 5 μm) using a prepared slide (F-14781, Life Technologies), in which F-actin was stained with Texas Red-X phalloidin (red) and α-tubulin was stained with a primary mouse antibody to bovine α-tubulin and a secondary BODIPY FL goat anti-mouse antibody (green). (C, D) Raw (C) and deconvolved (D) iSIM images of a microtubule (scale bar 500 nm, pixel size 56 nm), from the region marked (yellow rectangle) in the combined image. The apparent microtubule width (FWHM in *x*–*y*) in this case was 188 nm (raw) and 138 nm (deconvolved), measured at the yellow line.

The PSF of the UoL iSIM, using the 1.49 NA objective (Table 1), was measured using images of microtubules (Fig. 4), which provided sub-diffractive (width 25 nm) fluorescent features when illuminated at 488 nm (prepared slide F-14781, Life Technologies). The FWHM of each of ten microtubule images were measured in *x*–*y* and *z*. Images were taken at 50 nm steps in *z*.

To measure the FWHM of a microtubule image in *x*–*y*, the brightest slice within a microtubule image and the maximum intensity projection from all *z*-slices were obtained. The width of the brightest slice more directly describes the shape of the 3D PSF, while the maximum intensity projection reflects the appearance of images when this function is used to obtain a projection of in-focus information from a 3D *z-*stack. In each case, *ImageJ* was used to plot an intensity profile across the width of the microtubule, and the FWHM of the profile was found from a Gaussian fit, also performed in *ImageJ*. To measure the FWHM of a microtubule image in *z*, an *x*–*z* slice was taken through it, and the FWHM found from the profile of the slice in the *z*-direction, also using a Gaussian fit. Results from the raw iSIM images were used as PSF parameters in deconvolution for 40 iterations of the algorithm (i.e. in a 3D Gaussian PSF with FWHM of 4 × 4 × 9 pixels).

**Fig. 5:**
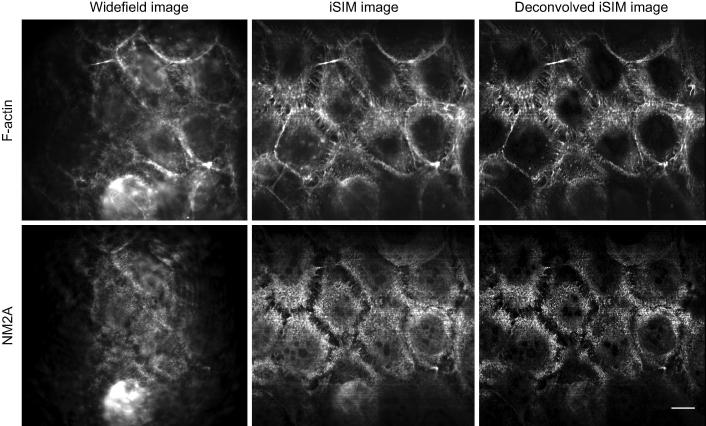
Widefield, iSIM and deconvolved images captured using the iSIM at Leeds, with a 60× water immersion objective lens (Table 1). Resolution increases when changing from widefield fluorescence mode (no lenslet or pinhole arrays in place) to iSIM, and further following image deconvolution. Scale bar 10 μm. Sample is BPH1 cells stained for F-actin (red), and non-muscle myosin 2A (green).

The PSF measured on our system was similar in size to that of the originally reported iSIM [14] (Table 3). Images acquired also clearly show the resolution improvement, compared with widefield microscopy (Fig. 5). The final resolution is lower than that achieved by 3D SIM, for instance (where FWHM in *x*–*y* ≈ 100 nm, in *z* ≈ 280 nm) [10], although both 3D SIM and iSIM attempt to achieve the theoretical twofold improvement in resolution over widefield microscopy. In fact, the iSIM and MSIM [13] do achieve an approximately twofold increase, but also start with a lower resolution in their widefield modes [13]. In the MSIM, this was partly assumed to be a result of slight curvature of an ideally flat optical surface; similarly in the iSIM, there are many optical components on the emission path, and slight imperfections or misalignments among them may prevent optimisation of the widefield resolution. In discussion of the MSIM [13], it was also suggested that the distribution of spatial frequencies used for illumination in many SIM implementations improved the signal to noise ratio at the highest frequencies, compared with iSIM and MSIM, where all frequencies permitted by the objective are used to generate the multi-point illumination.

### 3.2 Temporal characteristics and adjustment

The typical, default exposure time for the maximum FOV is 40 ms (2 sweeps of the scanning mirror at 20 ms per sweep). The number of sweeps (Fig. 3A) and the duration of a single sweep (through “Settings”, “Galvo Settings”) can be adjusted in the graphical user interface.

The brief exposure time makes the iSIM useful for capturing time-lapse images of cellular dynamics; blur as a result of motion during the exposure is reduced, compared with slowler technologies. Two-colour and 3D time-lapse sequences have been captured using the UoL iSIM showing the locations of protein EB1 during the growth of microtubules (Supplementary video 1) and mitosis (Supplementary video 2).

To achieve image acquisition at or beyond 100 frames per second, as reported [14], it is necessary to reduce the ROI on the camera, decreasing the time required to transfer each image to the computer. Capture of a one-colour, 2D time-lapse sequence at a rate of one frame every 8 ms can be achieved using the following settings: In “Exposure” (Fig. 3A), use 1 sweep. In “Delay” (Fig. 3C), use 0.00. In “Settings”, “Galvo mirror”, use a “Sweep time” of 5.2 ms and set “DAQ points per sweep” to 128. In “Settings”, “Camera ROI”, set “y0” to 761 and “y1” to 1400. The text file produced alongside each 2D time-lapse sequence or *z-*stack provides the camera trigger repetition time.

Using the default FOV, single-colour, 2D images can still be acquired at 75 frames per second, and two-colour images can be acquired at 38 frames per second. For comparison, using a commercial line-scanning confocal microscope or SIM system to acquire a single time-point with a similar FOV and bit-depth takes around 1 s per image, as does acquisition with commercial SIM systems. If different emission filters are required for the two colour channels in iSIM, an additional delay of a few hundred milliseconds is introduced for the switch, although for “zero” delay between images, the specified number of images is acquired first with only 561 nm excitation, then with only 488 nm excitation.

The iSIM captures *z*-stacks with an interval of about 10 ms between slices. Again, in two-colour 3D imaging, the two channels are captured at each *z*-position of the stage, before moving to the next position, if the same emission filter can be used for both channels. If the emission filter must be switched between the two channels, the whole stack is imaged with 561 nm excitation and then again with 488 nm excitation.

### 3.3 Limitations

The main limitation of the iSIM is the scan lines which appear in images, as a result of imperfectly smooth combination of the contracted images as they are scanned past one another (visible in Figs. 4, 5, Supplementary videos 1 and 2). The micro-optics are aligned as well as possible (Section 2.2.1), but the scan lines could not be completely removed. They can be mitigated by dividing raw iSIM images either by a flat reference field (as for Supplementary ideos 1 and 2) or dividing by the result of binning the image in horizontal lines [16]. More ideally, micro-optics may be designed that reduce the striping artefact by changing the spacing of the micro-optic elements or perhaps the aperture profile of the pinholes. A spinning disk-based configuration [14], [17] may also help with smooth combination of the scanned, contracted images, as well as with detection efficiency of the fluorescent emission.

Towards the top and bottom of the default FOV, the intensity of detected light becomes weaker (as in Fig. 5). This is a result of the beam size in this optical design, combined with a smaller number of image points being scanned across the camera chip at these extremes. The different number of image points being scanned at different vertical positions in the total image can also result in a change of appearance of the scan line artefacts with vertical position in the image.

Deconvolved images have a mottled noise component (Supplementary videos 1 and 2), using the algorithms tested so far (see Section 2.3.3; the Landweber algorithm in *DeconvolutionLab* was also tested). This may be a consequence of scan lines interfering with the raw image; further work is needed to explore the reduction of this noise.

Aberrations are present in the system as a result of the diverging beams passing obliquely through the thick dichroic mirror and compensator plate, even though they compensate each other for astigmatism to some degree. Other monochromatic aberrations, such as coma, are not compensated in the same way, and neither is chromatic aberration, as beamlets at 488 and 561 nm are separated transversely by the tilted silica plates and their optical dispersion. Such aberrations in the system could be overcome if this dichroic mirror could be placed instead in a collimated beam path, in a redesigned optical arrangement.

Finally, the iSIM is an optical system with many components, which combine to reduce the efficiency of fluorescence detection. Improvements on parts of the design are possible in this respect, for instance using the spinning disk-based design mentioned [17]. For a given sample, such improvements would allow time-lapse imaging to continue for longer before photo-bleaching occurs.

## 4 Conclusion

The combination of very rapid, super-resolution image acquisition and relatively low excitation intensities makes the iSIM useful for live-cell imaging. It provides a twofold improvement in resolution in 3D over widefield microscopy, with acquisition rates exceeding 100 frames per second, with two-colour images displayed in real time. It can be replicated as described, using commercially available hardware components and publicly available software.

## Figures and Tables

**Table 1:** List of components used in the iSIM.

Component	Manufacturer	Notes
Optical table: M-RS2000-48-8	Newport Spectra-Physics Ltd. www.newport.com	Table was electrically earthed
Legs: S-2000A-423.5
Pedestal posts, holders: ESK021A/M, ESK01/M, ESK03/M	Thorlabs www.thorlabs.de	Except where described, 1″ and 2″ diameter optics were mounted in these lens mounts
Lens mounts: LMR1, LMR2
Sample stage: RAMM-BASIC-DV frame, MIM-FC-FOCUS-K (for objective and folding mirror), PZ2300 piezoelectric *z*-stage MS-2000 XYZ piezo stage control system	Applied Scientific Instrumentation	These components make up the basic frame, motorised stage insert, and housing for objective and folding mirror
UK distributors: www.lifescienceimaging.co.uk	A tube lens may be supplied by default with the RAMM frame, but needs to be removed to allow use of the Edmund Optics tube lens listed below
sCMOS camera: pco.edge 4.2 (air cooled)	PCO	The camera was mounted on a translation stage (Thorlabs, PT1B/M) to set its *z*-position, on mounting adaptor PT101. The camera housing is tapped with an imperial hole; it was supported with an imperial pillar post and set screw (Thorlabs, RS1.5P and SS25S088) clamped to the translation stage
UK distributors: www.photonlines.co.uk
Excitation lasers: (561 nm and 488 nm wavelengths) Genesis MX488-1000 STM Genesis MX561-500 STM	Coherent www.coherent.com	Both have a maximum power 1.1 W), both clamped to the optical table
Dichroic mirror 1: LPD01-400-RU-25	Semrock www.semrock.com	For combination of excitation laser paths
UK Distributors www.laser2000.co.uk
Acousto-optic tunable filter (AOTF): AOTFnC-400.650-TN, with 8 channel controller MDS8C-B66-22-74.158)	AA Opto-electronic	The AOTF deflects the beams into and out of the system path as required
UK distributors: Photon Lines Ltd. www.photonlines.co.uk	M3 bolts and washers were used with M3-M6 thread adaptors (Thorlabs, AE3M6M) to fasten the AOTF to pillar pedestal posts
Power supply for AOTF: IPS 303DD and 25-pin D-Sub connector (e.g. Amphenol, L77DB25SST; L717DB25PST (with gender changer)	Isotech	The D-Sub connector is required to connect the AOTF to the control channels of the breakout box and to the power supply

Supplied by Radio Shack	NB, the power supply must supply more than the maximum specified current (0.9 A) during the warm-up period (a current limit at 1.5 A was sufficient)
Half-wave plates: WPH10M-488 and AHWP05M-600	Thorlabs	Mounted in rotation mounts (Thorlabs, RSP1C)
To maximise diffraction efficiency of the AOTF for the 488 nm and 561 nm lasers
Beam expander: ^∗^64-837, *f *= 45 mm; ^∗∗^AC254-400-A-ML, *f *= 400 mm	^∗^Edmund Optics www.edmundoptics.co.uk	The shorter focal length lens was mounted in a v-clamp (Thorlabs, VC3)
^∗∗^Thorlabs
Scan lens × 4: *f* = 190 mm, 55-S190-60-VIS	Special Optics www.specialoptics.com	Each mounted in a v-clamp (Thorlabs, VC3, with extension post MS1R/M)
Tube lens: 49-289-INK, *f *= 350 mm	Edmund Optics	This was mounted in a tip-tilt mount (Thorlabs KM200)
Objective lenses: APON60XOTIRF, NA 1.49, apochromatic TIRF lens	Olympus www.Olympus-lifescience.com	*f *= 3 mm
UPLSAPO60XS, NA 1.3, plan, super-apochromatic
UPLSAPO60XW, NA 1.2, plan, super-apochromatic
Relay lens x 2: AC508-300-A-ML, *f *= 300 mm	Thorlabs	
Dichroic mirror 2: 488-561 DM	Iridian Spectral Technologies www.iridian.ca	To separate emission and excitation light
UK distributors: Laser Lines www.laserlines.co.uk	Filter is 6 mm thick to reduce curvature of the reflecting surface introduced by clamping in its mount
Mount used was KM100C (Thorlabs)
Filter wheel: Lambda 10-B	Sutter Instrument Company www.sutter.com	To house filters for further rejection of excitation light and possible bandpass separation of emission channels
UK distributors: Photometrics www.photometrics.com
Notch filters: NF03-488E-25 and NF03-561E-25	Semrock www.semrock.com	For use in the filter wheel, for further rejection of excitation light
UK Distributors www.laser2000.co.uk
Scanning mirror: SPO9086, coated on both sides	Sierra Precision Optics www.sierraoptics.com	The scanning mirror was directly shipped to Nutfield Technologies for mounting
Mounting and control for Scanning Mirror: QS-12 based Single Axis Scan Set N-2071, with connector cables C-PWR-FL-36 and C-CMD-FL-36 and mounting block OFH-QS12-15	Nutfield Technologies www.nutfieldtech.com	The mounting block requires substantial modification for use in the iSIM (sawing in half and tapping threaded holes), since it is designed for two-axis scanning. An alternative should be considered
Power supply (±15 V) for Galvo scanning mirror control circuit board: 32212C (±15 V)	Calex www.calex.co.uk	Power supply requires a safety cover, insulating putty or other protection from the open contacts where the electrical mains is connected
It was necessary to earth the COM output of the power supply to achieve symmetry of the supply voltages relative to ground
Lenslet arrays: APO-Q-P222-F1.86 (*f *= 1.86 mm @633 nm)	Advanced Microoptic Systems www.amus.de	1 mm thick, comprising a square grid (pitch: 222 μm) of microlenses. Anti-reflection coated (400–650 nm)
APO-Q-P222-F0.93 (*f *= 0.93 mm @633 nm)	These were mounted in rotation, tip-tilt and translation mounts (Thorlabs: K6XS), and fixed to a further *z*-translation stage (MS1/M) via a pedestal pillar post on a magnetic removable base (Thorlabs: KB75/M), so that the arrays could be removed for some stages of alignment and testing
Pinhole array: 40 μm diameter pinholes, square array with pitch 222 μm, chrome on 0.090-inch-thick quartz)	Photosciences www.photosciences.com	Mounted in the same way as the lenslet arrays
Broadband AR coated back & front. (We also purchased an equivalent pinhole array with 50 μm diameter pinholes that might be more suitable for thin specimens)
Compensator plate: 0.025″ thick PW1-2025-UV	Melles Griot www.cvimellesgriot.com	
UK distributor: http://cvilaseroptics.com/
Plane mirrors: 1″ diameter × 7, KM100-E02, 2″ diameter × 5, KM200-E02, Periscope assembly, RS99/M	Thorlabs	These parts include tip-tilt mounts for mounting
Brightfield source	Office Depot	A standard anglepoise desk lamp was used during construction
Analogue output card: PCI-6733, with breakout box BNC-2110	National Instruments uk.ni.com/	Electronic control and image acquisition (from a Python script – see Sections 2.2.2 and 2.2.3)
This is different from the control arrangement of the previously published iSIM [14], which used a different analogue output card and required extra home-built amplifiers for the control currents
Computer for control and acquisition: Intel Core i7-3820 CPU, 64 GB RAM, 64-bit Windows 7	Stone http://www.stonegroup.co.uk/	
Shelf unit PTA278	Thorlabs	Overbench and underbench storage, with powerstrips

**Table 2:** Components used in iSIM alignment.

Component	Manufacturer	Notes
Neutral density filters:	Thorlabs	Used to attenuate the beams for some stages of alignment (e.g. when focussing beams onto a sensor)
NE10A-A (OD 1.0)
NE30A-A (OD 3.0)
Shearing interferometer: SI254, with additional shear plates SI035P and SI100P	Thorlabs	For testing beam collimation
Collimated diode laser: CPS532, with power supply LDS5-EC	Thorlabs	Provides an auxiliary beam for testing system alignment. Mounted in pitch/yaw mount KAD11F (Thorlabs)
Optical power meter kit: PM130D	Thorlabs	Kit includes an optical power sensor
Image acquisition software: CamWare	PCO	This is installed with the PCO Edge 4.2 camera; useful for some alignment checks and procedures
Colour CMOS camera: DCC1645C	Thorlabs	Auxiliary camera, useful for detecting intermediate images in the optical system (with accompanying software)

**Table 3:** Performance of the iSIM system at Leeds (UoL), in comparison with the previously published iSIM.

	Original iSIM [14] (NA = 1.45)		UoL iSIM (NA = 1.49)	
Raw	Deconvolved	Raw	Deconvolved
Lateral FWHM (nm)	213 ± 26^a^	145 ± 14	212 ± 25^s^	152 ± 13^s^
216 ± 19^m^	145 ± 9^m^
Axial FWHM (nm)	511 ± 24	356 ± 37	513 ± 33	320 ± 16

a Mean ± standard deviation.

s Width obtained from slice of best focus at the microtubule.

m Width obtained from maximum intensity projection of all slices onto the *x*–*y* plane.

## Appendix A Supplementary data

Supplementary note 1

Supplementary video 1 Two-colour time-lapse imaging. 2D (single *z*-slice) time-lapse image of eGFP-EB1 (green) and stained mitochondria (red) in a live COS7 cell. Scale bar 10 μm, 2 s delay between frames, 40 frames. Images were captured with a 60× silicone oil immersion objective (Table 1). To mitigate scan line artefacts, each raw image was divided by an in-focus reference image of a layer of fluorescein, using *Calculator Plus* in *FIJI* (J. Schindelin et al., Nat. Methods 9 (2012) 676-682), using the maximum pixel value in the reference image as a multiplier to normalise. Each slice was then deconvolved using *decon.py*. Bleach correction was carried out in *FIJI*, using the “Simple Ratio” method for green and the “Histogram Matching” method for red.

Supplementary video 2 3D time-lapse imaging. Maximum intensity projections derived from 3D (*z*-stack) time-lapse images of eGFP-EB1 in a mitotic COS7 cell. Scale bar 5 μm, 12 *z*-slices at 0.25 μm separation, 2 s delay between volumes, 18 volumes. Each volume was acquired in under 0.7 s; volume repetition time was 3 s. Images were captured with a 60× silicone oil immersion objective (Table 1). To mitigate scan line artefacts, each raw slice was divided by an in-focus reference image of a layer of fluorescein, using *Calculator Plus* in *FIJI*, using the maximum pixel value in the reference image as a multiplier to normalise. Each volume was then deconvolved using *decon.py*. Depth coding was performed using a modified version of *K_TimeRGBcolorcode.ijm* (*ImageJ* macro by Kota Miura, Centre for Molecular and Cellular Imaging, EMBL Heidelberg, Germany).

## Footnotes

1 Since Google Code is to be discontinued, the software will soon be moved to the GitHub hosting service. Please contact the authors for further information.

## References

[1] Hell S.W. (2007). Far-field optical nanoscopy. Science.

[2] Huang B., Babcock H., Zhuang X. (2010). Breaking the diffraction barrier: super-resolution imaging of cells. Cell.

[3] Gustafsson M.G. (2000). Surpassing the lateral resolution limit by a factor of two using structured illumination microscopy. J. Microsc..

[4] Hell S.W., Wichmann J. (1994). Breaking the diffraction resolution limit by stimulated emission: stimulated-emission-depletion fluorescence microscopy. Opt. Lett..

[5] Betzig E. (2006). Imaging intracellular fluorescent proteins at nanometer resolution. Science.

[6] Hess S.T., Girirajan T.P., Mason M.D. (2006). Ultra-high resolution imaging by fluorescence photoactivation localization microscopy. Biophys. J..

[7] Rust M.J., Bates M., Zhuang X. (2006). Sub-diffraction-limit imaging by stochastic optical reconstruction microscopy (storm). Nat. Methods.

[8] Gustafsson M.G. (2005). Nonlinear structured-illumination microscopy: wide-field fluorescence imaging with theoretically unlimited resolution. Proc. Natl. Acad. Sci. USA.

[9] Rego E.H. (2012). Nonlinear structured-illumination microscopy with a photoswitchable protein reveals cellular structures at 50-nm resolution. Proc. Natl. Acad. Sci. USA.

[10] Gustafsson M.G. (2008). Three-dimensional resolution doubling in wide-field fluorescence microscopy by structured illumination. Biophys. J..

[11] Sheppard C.J.R. (1988). Super-resolution in confocal imaging. Optik.

[12] Muller C.B., Enderlein J. (2010). Image scanning microscopy. Phys. Rev. Lett..

[13] York A.G. (2012). Resolution doubling in live, multicellular organisms via multifocal structured illumination microscopy. Nat. Methods.

[14] York A.G. (2013). Instant super-resolution imaging in live cells and embryos via analog image processing. Nat. Methods.

[15] W.S. Rasband, Imagej, U.S. National Institutes of Health, Bethesda, Maryland, USA. <http://imagej.nih.gov/ij/> (accessed 10.03.2015).

[16] Winter P.W. (2015). Incoherent structured illumination improves optical sectioning and contrast in multiphoton super-resolution microscopy. Opt. Express.

[17] Azuma T., Kei T. (2015). Super-resolution spinning-disk confocal microscopy using optical photon reassignment. Opt. Express.

