# Supplementary material for: Construction of an instant structured illumination microscope
Source: Methods. 2015 Oct 15;88:37–47. doi: 10.1016/j.ymeth.2015.07.012 (PMC4641873; doi:10.1016/j.ymeth.2015.07.012)
Supplement: Supplementary note 1 [file mmc1.pptx]

## Slide 1
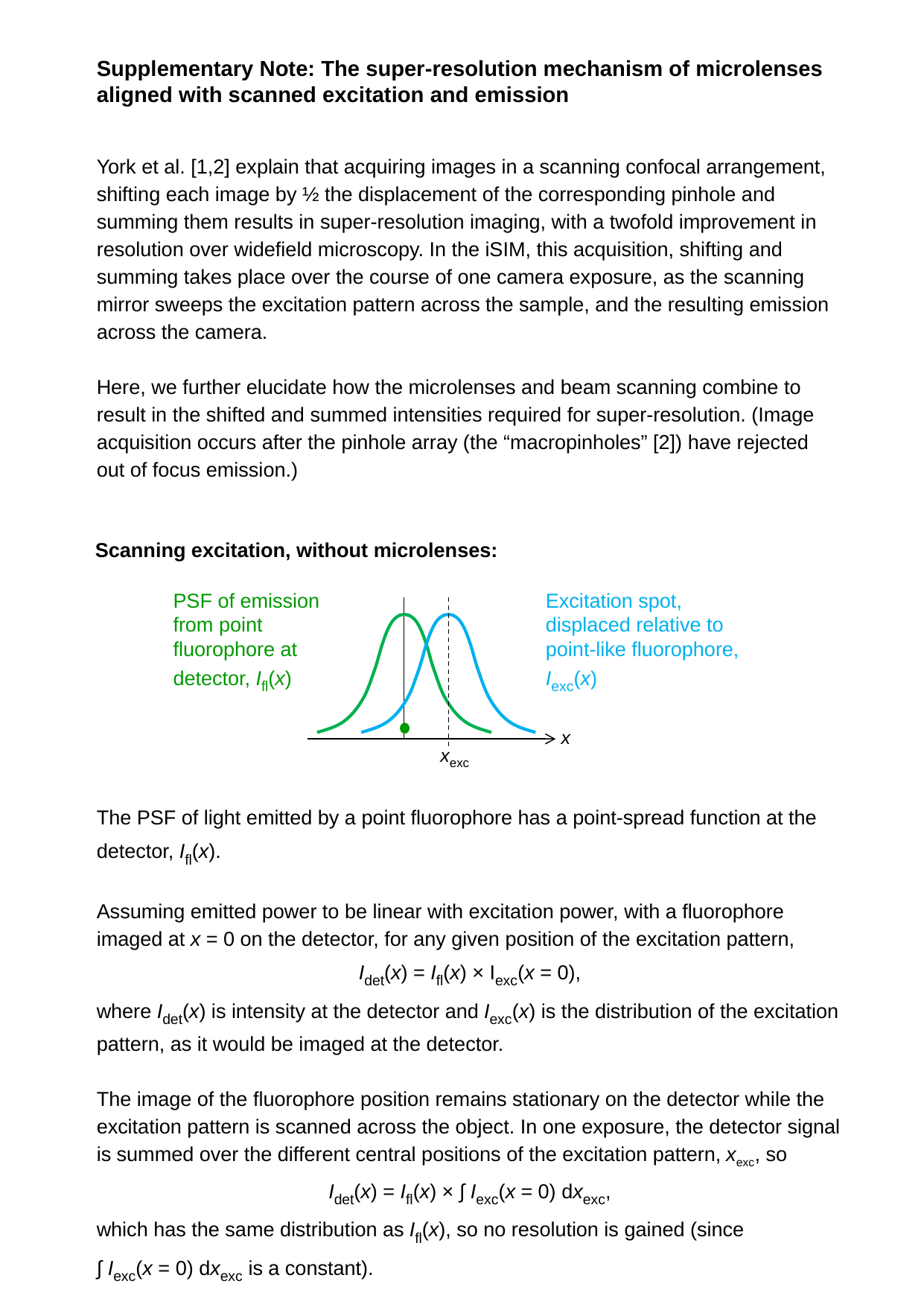

Supplementary Note: The super-resolution mechanism of microlenses aligned with scanned excitation and emission
York et al. [1,2] explain that acquiring images in a scanning confocal arrangement, shifting each image by ½ the displacement of the corresponding pinhole and summing them results in super-resolution imaging, with a twofold improvement in resolution over widefield microscopy. In the iSIM, this acquisition, shifting and summing takes place over the course of one camera exposure, as the scanning mirror sweeps the excitation pattern across the sample, and the resulting emission across the camera.
Here, we further elucidate how the microlenses and beam scanning combine to result in the shifted and summed intensities required for super-resolution. (Image acquisition occurs after the pinhole array (the “macropinholes” [2]) have rejected out of focus emission.)
Scanning excitation, without microlenses:
Excitation spot,
displaced relative to
point-like fluorophore,
Iexc(x)
PSF of emission from point fluorophore at detector, Ifl(x)
x
xexc
The PSF of light emitted by a point fluorophore has a point-spread function at the detector, Ifl(x).
Assuming emitted power to be linear with excitation power, with a fluorophore imaged at x = 0 on the detector, for any given position of the excitation pattern,
Idet(x) = Ifl(x) × Iexc(x = 0),
where Idet(x) is intensity at the detector and Iexc(x) is the distribution of the excitation pattern, as it would be imaged at the detector.
The image of the fluorophore position remains stationary on the detector while the excitation pattern is scanned across the object. In one exposure, the detector signal is summed over the different central positions of the excitation pattern, xexc, so
Idet(x) = Ifl(x) × ∫ Iexc(x = 0) dxexc,
which has the same distribution as Ifl(x), so no resolution is gained (since
∫ Iexc(x = 0) dxexc is a constant).

## Slide 2
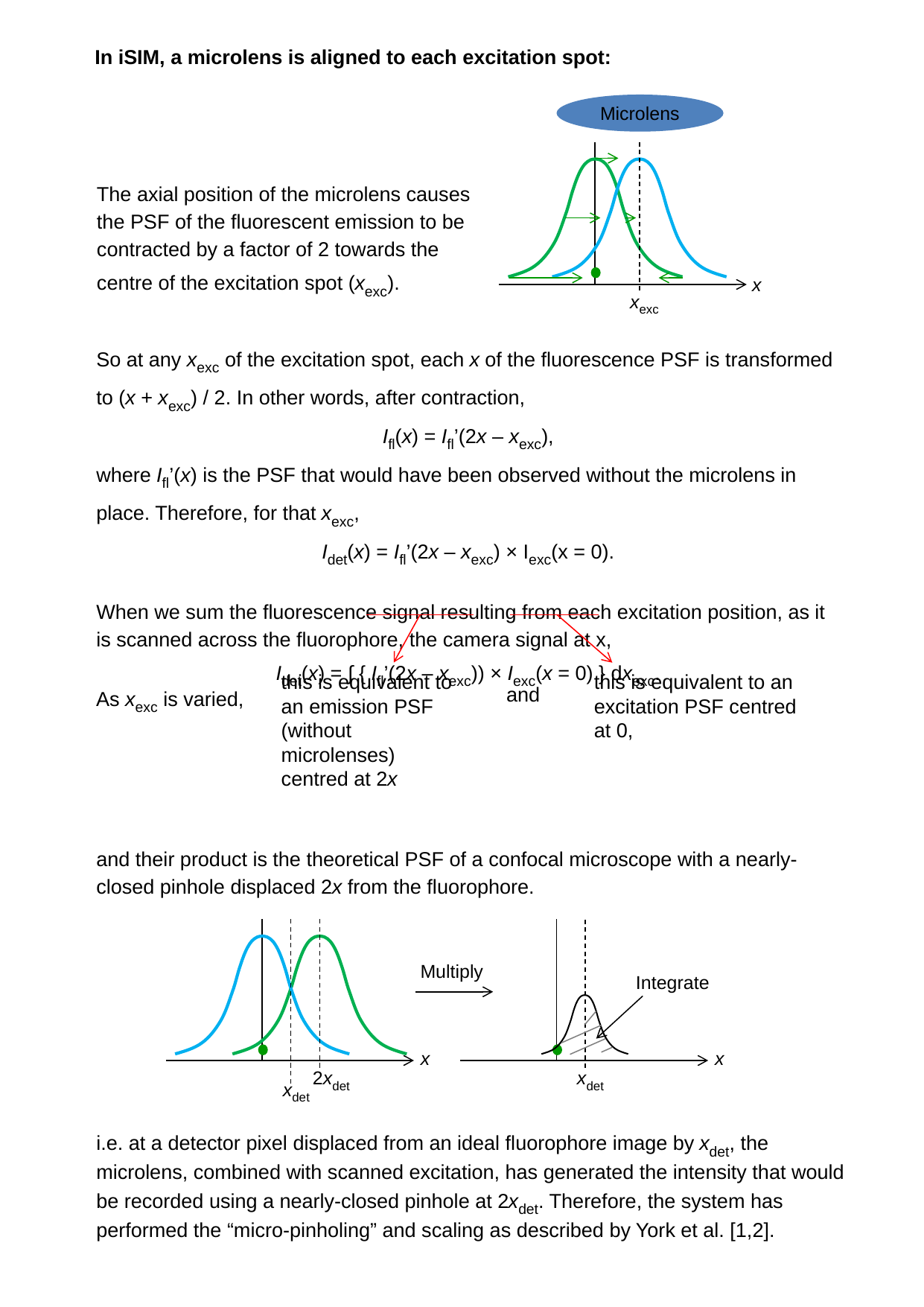

In iSIM, a microlens is aligned to each excitation spot:
Microlens
The axial position of the microlens causes the PSF of the fluorescent emission to be contracted by a factor of 2 towards the centre of the excitation spot (xexc).
x
xexc
So at any xexc of the excitation spot, each x of the fluorescence PSF is transformed to (x + xexc) / 2. In other words, after contraction,
Ifl(x) = Ifl’(2x – xexc),
where Ifl’(x) is the PSF that would have been observed without the microlens in place. Therefore, for that xexc,
Idet(x) = Ifl’(2x – xexc) × Iexc(x = 0).
When we sum the fluorescence signal resulting from each excitation position, as it is scanned across the fluorophore, the camera signal at x,
Idet(x) = ∫ { Ifl’(2x – xexc)) × Iexc(x = 0) } dxexc.
this is equivalent to an emission PSF (without microlenses) centred at 2x
this is equivalent to an
excitation PSF centred at 0,
As xexc is varied,
and
and their product is the theoretical PSF of a confocal microscope with a nearly-closed pinhole displaced 2x from the fluorophore.
Multiply
Integrate
x
x
2xdet
xdet
xdet
i.e. at a detector pixel displaced from an ideal fluorophore image by xdet, the microlens, combined with scanned excitation, has generated the intensity that would be recorded using a nearly-closed pinhole at 2xdet. Therefore, the system has performed the “micro-pinholing” and scaling as described by York et al. [1,2].

## Slide 3
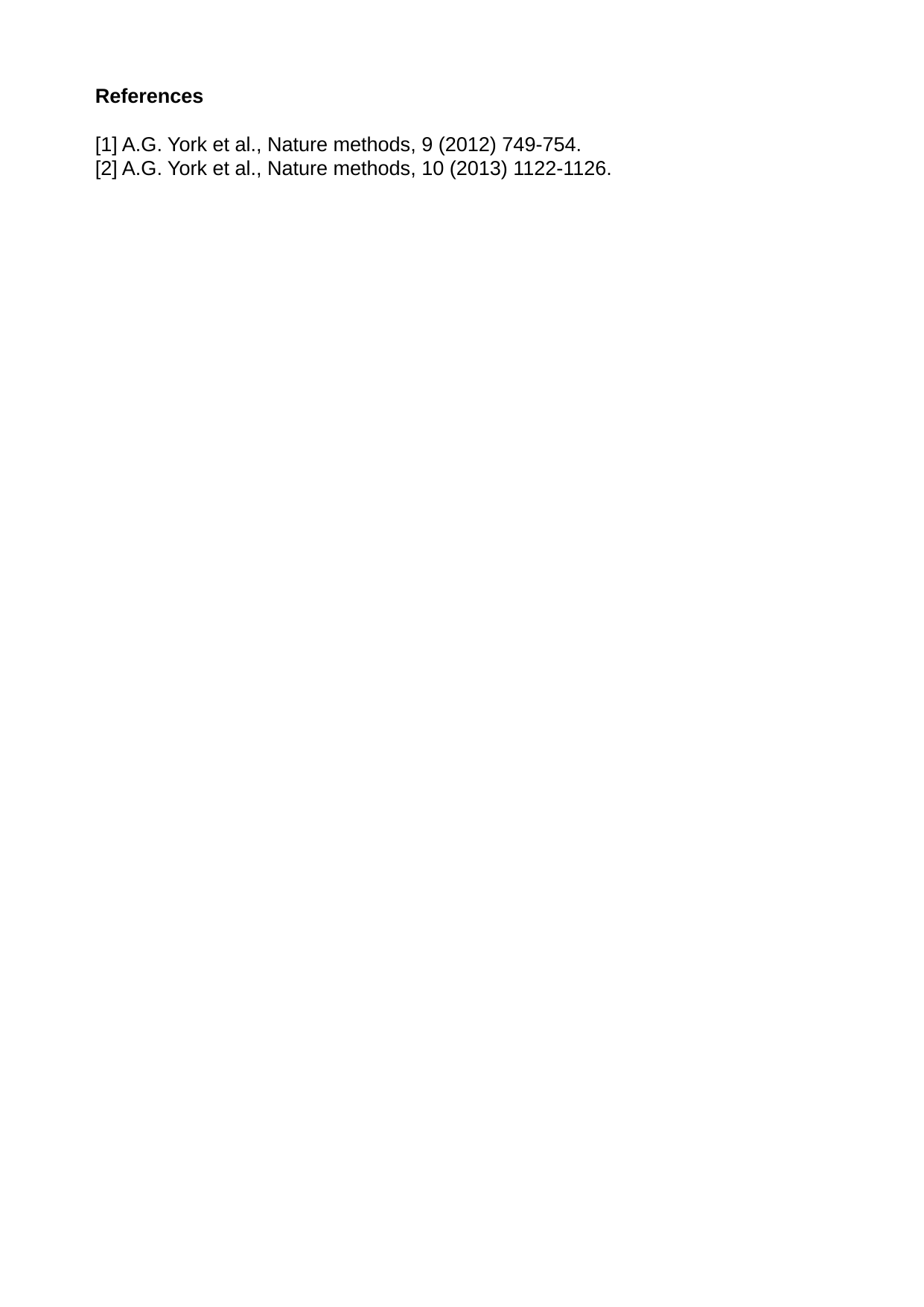

References
[1] A.G. York et al., Nature methods, 9 (2012) 749-754.
[2] A.G. York et al., Nature methods, 10 (2013) 1122-1126.
