# Supplementary figures and images for: Construction of an instant structured illumination microscope
Source: Methods. 2015 Oct 15;88:37–47. doi: 10.1016/j.ymeth.2015.07.012 (PMC4641873; doi:10.1016/j.ymeth.2015.07.012)

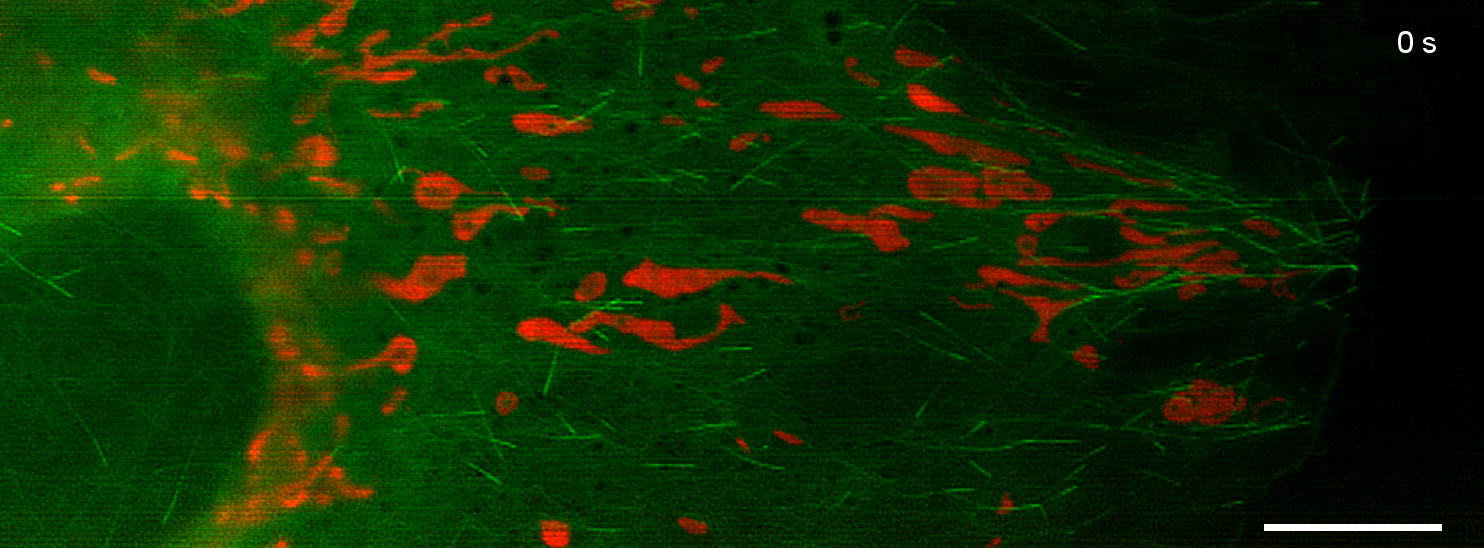

Supplement: Supplementary video 1 — Two-colour time-lapse imaging. 2D (single z-slice) time-lapse image of eGFP-EB1 (green) and stained mitochondria (red) in a live COS7 cell. Scale bar 10 μm, 2 s delay between frames, 40 frames. Images were captured with a 60× silicone oil immersion objective (Table 1). To mitigate scan line artefacts, each raw image was divided by an in-focus reference image of a layer of fluorescein, using Calculator Plus in FIJI (J. Schindelin et al., Nat. Methods 9 (2012) 676-682), using the maximum pixel value in the reference image as a multiplier to normalise. Each slice was then deconvolved using decon.py. Bleach correction was carried out in FIJI, using the “Simple Ratio” method for green and the “Histogram Matching” method for red. [file mmc2.jpg]

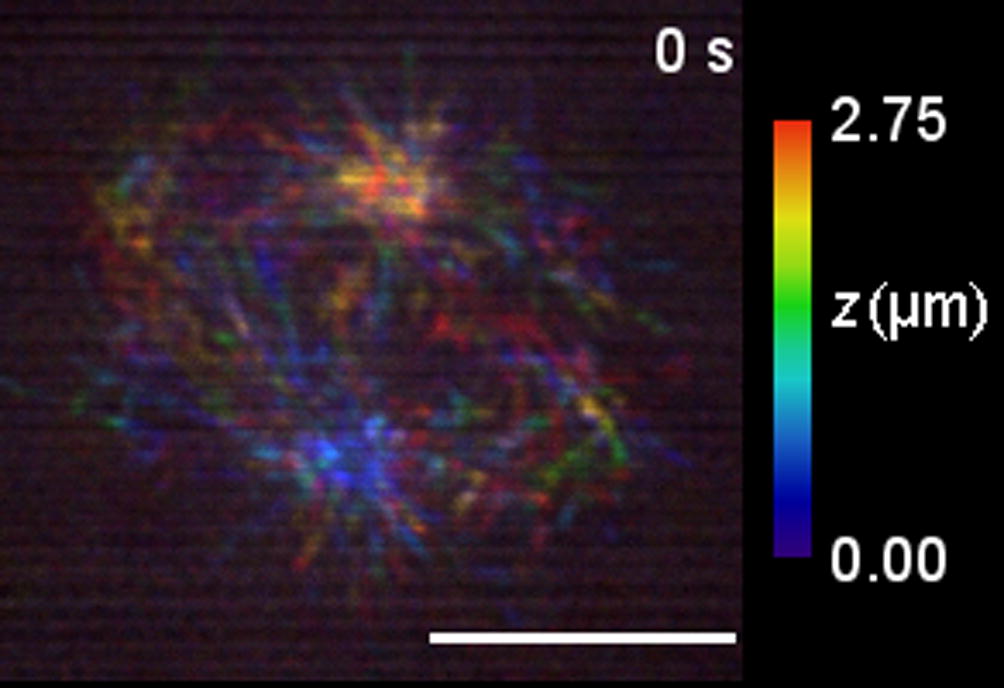

Supplement: Supplementary video 2 — 3D time-lapse imaging. Maximum intensity projections derived from 3D (z-stack) time-lapse images of eGFP-EB1 in a mitotic COS7 cell. Scale bar 5 μm, 12 z-slices at 0.25 μm separation, 2 s delay between volumes, 18 volumes. Each volume was acquired in under 0.7 s; volume repetition time was 3 s. Images were captured with a 60× silicone oil immersion objective (Table 1). To mitigate scan line artefacts, each raw slice was divided by an in-focus reference image of a layer of fluorescein, using Calculator Plus in FIJI, using the maximum pixel value in the reference image as a multiplier to normalise. Each volume was then deconvolved using decon.py. Depth coding was performed using a modified version of K_TimeRGBcolorcode.ijm (ImageJ macro by Kota Miura, Centre for Molecular and Cellular Imaging, EMBL Heidelberg, Germany). [file mmc3.jpg]
